# Biodegradable Poly(butylene adipate-*co*-terephthalate) Antibacterial Nanocomposites Reinforced with MgO Nanoparticles

**DOI:** 10.3390/polym13040507

**Published:** 2021-02-08

**Authors:** Xionggang Wang, Lingna Cui, Shuhong Fan, Xia Li, Yuejun Liu

**Affiliations:** Key Laboratory of Advanced Packaging Materials and Technology of Hunan Province, School of Packaging and Materials Engineering, Hunan University of Technology, Zhuzhou, Hunan 412007, China; cheersun@126.com (X.W.); lncui@hut.edu.cn (L.C.); shfan@hut.edu.cn (S.F.); xiali_2019@163.com (X.L.)

**Keywords:** PBAT, MgO, thermal stability, antibacterial, mechanical property

## Abstract

Antibacterial packaging materials can reduce the microbial contamination of food surfaces. In this study, magnesium oxide (MgO) nanoparticles were synthesized and then coated with cetrimonium bromide (CTAB). CTAB-modified MgO (MgO@CTAB) was characterized by Fourier-transform infrared spectroscopy (FT-IR), X-ray diffraction (XRD), and thermogravimetric analysis. Then, different loadings of MgO@CTAB were mixed with poly(butylene adipate-co-terephthalate) (PBAT) by melt compounding. The results showed that the addition of MgO@CTAB deteriorated the thermal stability of PBAT due to MgO serving as a catalyst to promote the thermal degradation of PBAT. In addition, MgO@CTAB could serve as a nucleating agent to improve the crystallinity of PBAT. With the optimal 3 wt% of MgO@CTAB, the tensile strength of PBAT/MgO@CTAB increased from 26.66 to 29.90 MPa, with a slight enhancement in elongation at break. SEM observations and dynamical rheological measurements revealed that aggregation occurred when the content of MgO@CTAB exceeded 5 wt%. The presence of MgO@CTAB endowed PBAT with antibacterial properties. The bacterial inhibition zone increased with the increasing content of MgO@CTAB. In addition, MgO@CTAB had a better antibacterial efficiency against Gram-positive bacterial *S. aureus* than Gram-negative bacterial *E. coli.*

## 1. Introduction

Biodegradable food packaging polymers have gained considerable interest from both academia and industry due to the depletion of petroleum resources and the pressure of environmental protection. Poly(butylene adipate-co-terephthalate) (PBAT) is a type of aliphatic aromatic copolyester thermoplastic, which can be synthesized by the copolymerization of the monomers 1,4-butanediol, adipic acid, and terephthalic acid [1]. PBAT exhibits good biodegradability, high flexibility, excellent tear resistance, and good processability [2,3]. It has been widely utilized for different packaging applications, such as garbage bags, solid food containers, and film wraps. To fulfill requirements for the freshness, safety, and quality of foods, there is growing demand for antibacterial packaging materials in food packaging sectors [4,5,6]. Various types of metal oxides, such as zinc oxide (ZnO), silver oxide (Ag_2_O), magnesium oxide (MgO), and titanium oxide (TiO_2_), have been developed and exhibited different degrees of antibacterial effects on PBAT composites [7,8,9].

Magnesium oxide (MgO) can be easily synthesized from magnesium-rich minerals. It has been considered a promising alternative antibacterial agent due to its environmental friendliness, non-toxicity, low cost, and illumination independence [10,11]. Moreover, MgO has been listed as a safe material by the U.S. Food and Drug Administration (US-FDA). It has been proposed that the presence of abundant defects or oxygen vacancy on the surface of MgO nanoparticles will contribute to the generation of reactive oxygen species (ROS), which can destroy bacterial cells and protein peptides [12]. Wang et al. prepared nano–MgO/carboxymethyl chitosan (CMCS) films using a solution method [13]. The results revealed that the introduction of MgO contributed to the improvement of thermal stability, UV shielding performance, and water resistance of CMCS films. The CMCS films increased elasticity and ductility simultaneously with the addition of 1 wt% of MgO nanoparticles. Swaroop and his colleague demonstrated that the tensile strength and oxygen barrier properties of polylactide (PLA) composite films containing 2 wt% of MgO increased by 29% and 25%, respectively, as compared with those of pure PLA films [14]. In addition, PLA/MgO films also exhibited pronounced inhibition of *E. coli* bacterial [15].

Cetyltrimethylammonium bromide (CTAB) is a quaternary ammonium surfactant that has strong inhibitory effects on a variety of bacteria through electrostatic interactions that disrupt or disturb the cell membrane [16,17]. This inexpensive cationic surfactant is safe at low concentrations. Chang et al. utilized CTAB to modify Janus silver mesoporous silica nanoparticles using a one-pot method [17]. The minimum inhibitory concentrations (MIC) for *S. aureus* and *E. coli* were determined to be 20 and 10 μg/mL, respectively. Sasikumar et al. found that the presence of CTAB could not assist the synthesis of BaTiO_3_ nanoparticles via a hydrothermal method, but it endowed BaTiO_3_ nanoparticles with antimicrobial properties [18]. The CTAB-functionalized BaTiO_3_ exhibited profound antibacterial behaviors against *C. albicans* bacteria. To date, to the best of our knowledge, the application of MgO nanoparticles in combination with CTAB as a bactericide for PBAT has not yet been explored.

In this work, MgO nanoparticles were synthesized using a sol–gel method. Then, CTAB-functionalized MgO nanoparticles (MgO@CTAB) were incorporated into the PBAT matrix to prepare antibacterial nanocomposites. The effects of MgO@CTAB loading on the thermal stability, crystallization behavior, rheological and mechanical properties, as well as the antibacterial performance of PBAT nanocomposites were investigated.

## 2. Materials and Methods

### 2.1. Materials

Poly(butylene adipate-co-terephthalate) (PBAT) with a melt index of 5 (190 °C, 2.16 kg) was supplied by BASF (Shanghai, China). Magnesium chloride hexahydrate (MgCl_2_·6H_2_O), sodium hydroxide (NaOH), and cetyltrimethylammonium bromide (CTAB) were obtained from J&K (Shanghai, China).

### 2.2. Preparation of MgO@CTAB

Magnesium oxide (MgO) nanoparticles were prepared using a modified sol–gel method [19]. First, 0.5 g of CTAB and 1.6 g of MgCl_2_·6H_2_O were added to 40 mL of DI water with vigorous stirring for 2 h to form clear gel. A 20 mL solution of 0.4 M NaOH was slowly added to the as-obtained gel. Then, the mixture was transformed into a 100 mL PPL-lined stainless steel autoclave and kept at 120 °C overnight. The obtained precipitate was centrifuged with DI water and ethanol twice and dried at 60 °C for 6 h. The resulting powders were calcinated in a tubular furnace at 400 °C for 3 h. Two grams of the as-prepared MgO nanoparticles and 1.0 g of CTAB were dispersed in 100 mL of ethanol with ultrasonication for 30 min. Then, the CTAB-modified MgO nanoparticles (MgO@CTAB) were centrifuged and rinsed by ethanol twice to remove the excess CTAB.

### 2.3. Preparation of PBAT/MgO@CTAB Nanocomposites

PBAT nanocomposites containing MgO@CTAB nanoparticles were prepared using the melt compounding method. The desired amount of MgO@CTAB was mixed with PBAT in a Brabender internal mixer (Brabender GmbH & Co. KG, Duisburg, Germany) at a fixed speed of 60 rpm at 160 °C for 8 min. Then, the samples were hot-compressed into specimens with different dimensions for characterization. The as-prepared PBAT/MgO@CTAB nanocomposites were abbreviated as MgO@CTAB-X, where X stands for the weight percentage of MgO@CTAB in the nanocomposite. For comparison, pristine MgO nanoparticles were also mixed with PBAT using the same procedures.

### 2.4. Characterization

The morphology and structure of MgO@CTAB were investigated by transmission electron microscopy (TEM, JEOL JEM-2100F, Tokyo, Japan) and scanning electron microscopy (SEM, FEI 250, FEI Company, Hillsboro, OR, USA) with energy-dispersive X-ray (EDX) spectroscopy. The MgO@CTAB and cryo-fracture surfaces of PBAT/MgO@CTAB nanocomposites were sputtered with a thin layer of gold before SEM observation. Fourier-transform infrared spectroscopy (FT-IR) spectra were collected on a Nicolet NEXUS670 spectrometer (Thermo Fisher Scientific Inc., Dreieich, Germany). The samples were scanned 16 times in the range of 400–4000 cm^−1^. The thermal stability was measured by a Netzsch TGA-209F1 thermogravimetric analyzer (TGA, NETZSCH GmbH & Co.Holding KG, Selb, Germany) under a nitrogen atmosphere. The specimens were heated from 30 to 700 °C at a ramping rate of 10 °C min^−1^. The X-ray diffraction (XRD) patterns were collected from 10 to 60° by using a Bruker D8 diffractometer (Bruker Daltonik GmbH, Bremen, Germany) with a fixed step of 0.02°. The crystallization and melting behavior of the PBAT composites were investigated using a Netzsch DSC-214 differential scanning calorimeter (DSC, NETZSCH-Gerätebau GmbH, Selb, Germany) in the range of 30–160 °C. The dynamical rheological performance was analyzed on an Anton Paar MCR302 rheometer (Anton Paar Australia Pty. Ltd., Sydney, Australia). The frequency sweep was conducted at 150 °C at a fixed strain of 1%. The tensile properties were measured on an Instron 5566 electron universal testing machine (Instron, Norwood, MA, USA). The tensile speed was fixed at 50 mm min^−1^. The reported results are the mean values of five successful specimens.

### 2.5. Antibacterial Properties

The antibacterial activity of the PBAT nanocomposites was investigated using the zone inhibition method [20]. In brief, a specific amount of bacterial culture (i.e., 0.1 mL) was transferred to nutrient agar plates. Then, 5 mm diameter circular films were put over the bacteria colonies and then incubated at 37 °C for 24 h to measure the bacterial inhibition zone.

## 3. Results and Discussions

**Figure 1:**
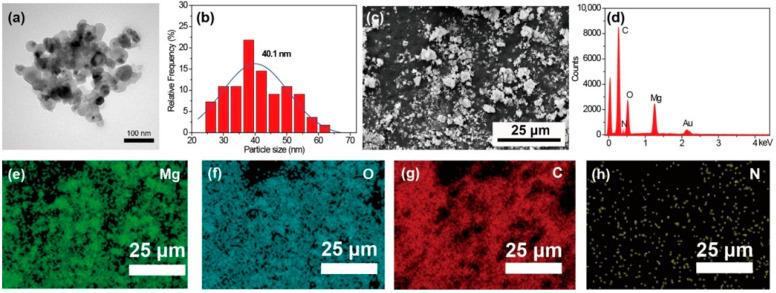
(**a**) Transmission electron microscopy (TEM) image; (**b**) particle size distribution of MgO; (**c**) scanning electron microscopy (SEM) image of MgO@CTAB; (**d**) energy-dispersive X-ray (EDX) result; EDX mapping element of (**e**) Mg, (**f**) O, (**g**) C, and (**h**) N.

The morphology of the MgO nanoparticles is shown in Figure 1a. The obtained MgO exhibits a roughly spherical structure. The diameter distribution calculated from TEM images is shown in Figure 1b. The average diameter of the MgO calculated using ImageJ software is 40.1 nm. The SEM image of MgO@CTAB is shown in Figure 1c, and the corresponding EDX result in Figure 1d confirms the chemical composition of MgO@CTAB, which has two sharp signal peaks that are attributed to magnesium and oxygen elements. The strong carbon signal is mainly due to carbon conductive paste. In addition, the signal peaks for nitrogen are observed, which may result from the presence of grafted CTAB. The EDX mapping images in Figure 1e–h indicate that the CTAB was coated on the surface of MgO@CTAB uniformly.

**Figure 2:**
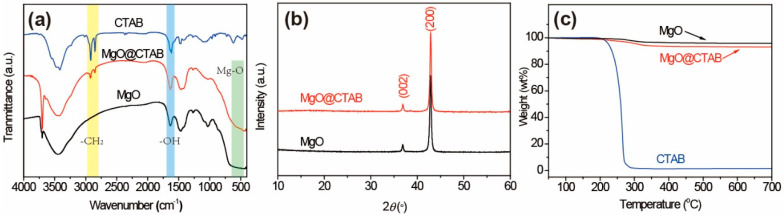
(**a**) Fourier-transform infrared (FT-IR) spectra, (**b**) XRD patterns, and (**c**) thermogravimetric analysis (TGA) curves of MgO and MgO@CTAB.

The FT-IR spectra of MgO and MgO@CTAB are shown in Figure 2a. The broad peaks at 3000–3700 cm^−1^ correspond to the stretching vibration of –OH groups, while the peaks at 1636 cm^−1^ represent the bending vibration of –OH groups due to the absorbed water molecules on the surfaces of the nanoparticles [21]. The detected peaks located at around 500 cm^−1^ are attributed to the stretching vibration of Mg-O bonds [22]. In addition, it is observed that two new peaks appear in MgO@CTAB at 2850 and 2922 cm^−1^, which are related to the asymmetric and symmetric stretching vibration of -CH_2_ bonds of the coated CTAB chains, respectively [23,24]. The above results indicate that the CTAB was coated on the surface of MgO successfully. The structures of MgO and MgO@CTAB are further confirmed by XRD patterns, as shown in Figure 2b. It is observed that the pure MgO nanoparticles have strong peaks located at 37.0° and 42.9°, which correspond to the (002) and (200) planes of JCPDS PDF-87-0652, respectively. After the decoration of CTAB, the MgO@CTAB exhibits similar absorption peaks as pure MgO, indicating that the addition of CTAB has no effect on the crystal structure of MgO. Figure 2c shows the thermal degradation behaviors of MgO and MgO@CTAB. It is observed that the pure MgO nanoparticles show 2.4 wt% loss in the temperature range of 250–340 °C, while a significant weight loss for MgO@CTAB occurs at temperatures from 200 to 350 °C. In addition, the char yields at 700 °C for MgO and MgO@CTAB are 95.8 wt% and 93.1 wt%, respectively, indicating that the coated CTAB in MgO@CTAB is around 2.7 wt%.

**Figure 3:**
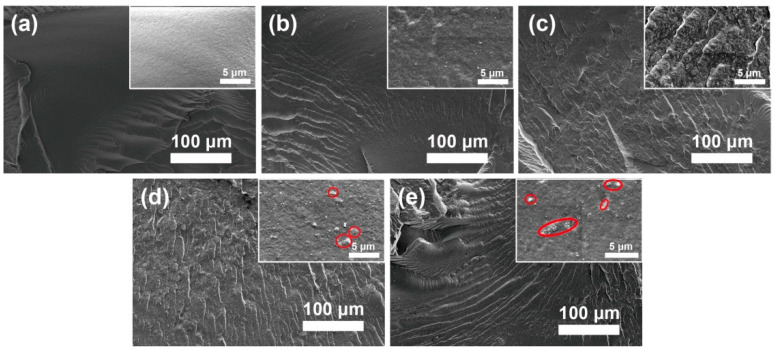
Fracture surface SEM images of poly(butylene adipate-co-terephthalate) (PBAT) nanocomposites. (**a**) MgO@CTAB-0, (**b**) MgO@CTAB-1, (**c**) MgO@CTAB-3, (**d**) MgO@CTAB-5, and (**e**) MgO@CTAB-7.

The fracture surface morphology of the PBAT nanocomposite was evaluated by using SEM, as shown in Figure 3. It is observed that MgO@CTAB-0 in Figure 3a exhibits a smooth surface due to its brittle fracture of PBAT. In addition, the fracture surfaces of the PBAT nanocomposites become rougher (Figure 3b–e) with the increasing MgO@CTAB loading, indicating that the presence of rigid MgO@CTAB nanoparticles contributes to the stress transformation [25]. Some aggregations (red circles) are observed on the fracture surface in the high-resolution SEM images when the content of MgO@CTAB is increased to 5 wt%.

**Figure 4:**
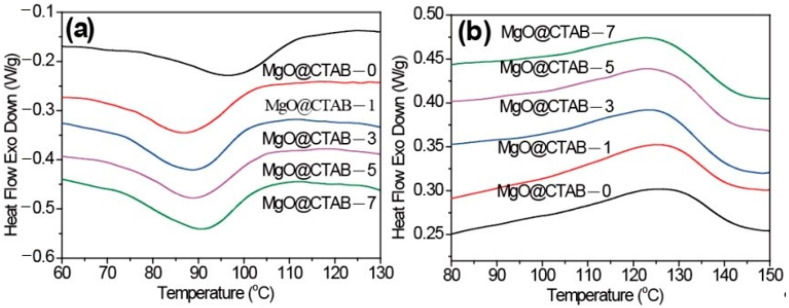
Differential scanning calorimetry (DSC) thermograms of PBAT/MgO@CTAB nanocomposites. (**a**) First cooling, (**b**) second heating.

The melting and crystallization behaviors of pure PBAT and its nanocomposites are shown in Figure 4. The peak crystallization temperature (*T*_c_), crystallization enthalpy (Δ*H*_c_), the peak melting temperature (*T*_p_), the melting enthalpy (Δ*H*_m_), and the crystallinity (*χ*) are summarized in Table 1. The degree of crystallinity was calculated by the following equation:

(1) $$\chi =\frac{∆H_{m}}{\left(1-W_{f}\right)∆H_{0}}$$

where Δ*H*_m_ represents to the melting enthalpy, *W*_f_ is the weight ratio of MgO@CTAB, and Δ*H*_0_ stands for the 100% melting enthalpy of PBAT (114 J g^−1^) [26].

In Figure 4a, it is observed that the initial crystallization temperatures and *T*_c_ of PBAT nanocomposites shift to lower values. Meanwhile, the Δ*H*_c_ values of PBAT nanocomposites increase slightly as compared with those of pure PBAT. These results indicate that the presence of MgO@CTAB contributes to the inhibition of crystal nucleation during the crystallization process. The *T*_p_ of PBAT nanocomposites in Figure 4b exhibits a decreasing trend with the increasing content of MgO@CTAB. Moreover, the crystallinity values of PBAT nanocomposite are all higher than those of pure PBAT.

**Figure 5:**
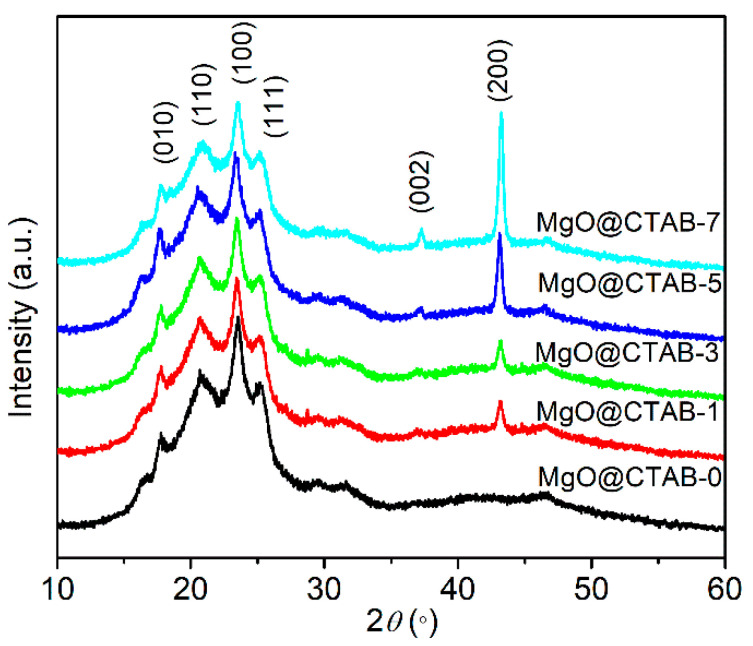
XRD patterns of PBAT nanocomposites with different loadings of MgO@CTAB.

The XRD patterns of PBAT nanocomposites are shown in Figure 5. Four diffraction peaks at 17.4°, 20.4°, 23.3°, and 25.1° are observed in MgO@CTAB-0, which correspond to the (010) (110) (100) and (111) planes of PBAT, respectively [27]. Furthermore, the intensity of the characteristic peaks at 37.3° and 43.2° gradually increases as the content of MgO@CTAB increases from 1 to 7 wt%, which are related to the (002) and (200) planes of MgO. In addition, it is noted that there is no significant shift or change in the diffraction peaks between pure PBAT and its nanocomposites.

**Figure 6:**
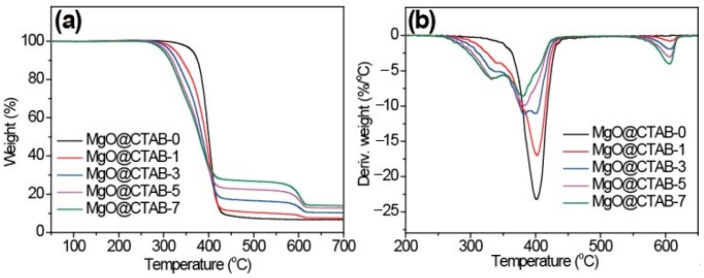
TGA curves of PBAT/MgO@CTAB nanocomposites. (**a**) TGA, (**b**) DTG.

The effects of MgO@CTAB on the thermal stability of PBAT were investigated through TGA, and the results are shown in Figure 6. The initial decomposition temperatures (*T*_10_), peak decomposition temperatures (*T*_p_), and char yields at 700 °C are listed in Table 2. In Figure 6, it is observed that the decomposition curves shift to lower temperatures with the increasing loading of MgO@CTAB. The *T*_10_ of MgO@CTAB-7 decreases from 375.2 to 313.7 °C as compared with that of MgO@CTAB-0. In addition, the values of *T*_p_ exhibit a similar decreasing trend. The decrease in thermal stability is mainly attributed to the catalytic effects of MgO, which is consistent with other reports [28]. Furthermore, the thermal decomposition of coated CTAB also resulted in weight loss. As expected, the char yields of PBAT nanocomposites at 700 °C increase gradually with the increasing content of MgO@CTAB.

**Figure 7:**
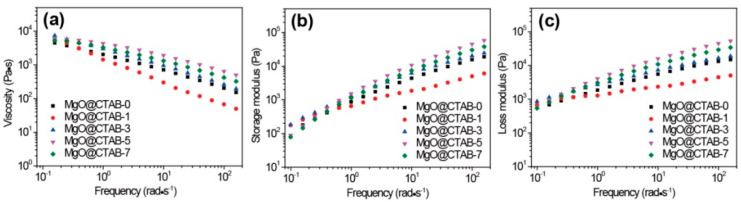
Dynamic rheological properties of PBAT nanocomposites with various contents of MgO@CTAB. (**a**) Complex viscosity, (**b**) storage modulus, and (**c**) loss modulus as a function of frequency.

The effects of MgO@CTAB on the rheological properties of PBAT were evaluated by taking dynamic rheological measurements. The complex viscosity (η*), storage modulus (*G*′), and loss modulus (*G*″) as a function of frequency at 150 °C are shown in Figure 7. In Figure 7a, the η* of all samples exhibits a typical shear thinning behavior. It is worth noting that the values of η* and *G*′ of MgO@CTAB-1 decrease significantly as compared with those of pure PBAT, which is consistent with the reported PBAT composite [29]. This may be ascribed to the presence of CTAB because it is a small molecule that improves the flowability of PBAT. The storage modulus of the PBAT nanocomposite in Figure 7b increases with the increasing content of MgO@CTAB. However, the enhancement of the storage modulus is not proportional to the content of MgO@CTAB. Enhancement of the storage modulus reaches its maximum value at 5 wt% of MgO@CTAB and then decreases with further loadings. This is due to the fact that the enhancement of MgO@CTAB is more profound than the inverse effect of the aggregation of MgO@CTAB in the PBAT matrix at the loading of 5 wt% MgO@CTAB. Figure 7c shows that the values of *G*″ decrease concomitantly with *G*′, indicating that the structure of the PBAT nanocomposites is most affected by filler content.

**Figure 8:**
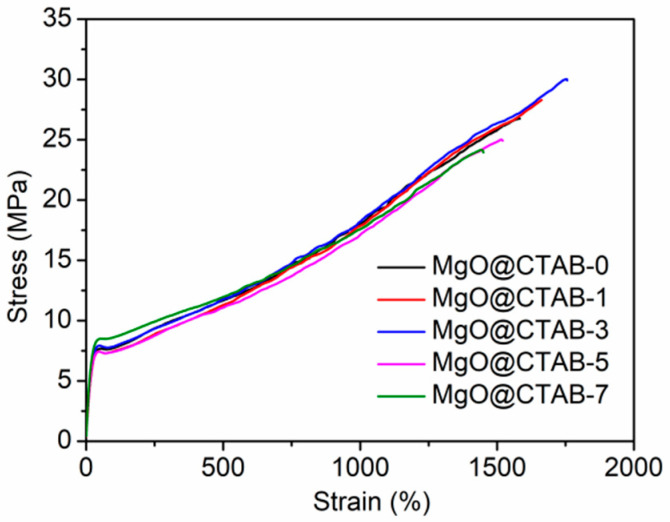
Tensile stress versus strain of PBAT nanocomposites.

The typical strain versus stress curves of PBAT and its nanocomposites are presented in Figure 8. The tensile stress, Young’s modulus, and elongation at break are summarized in Table 3. It can be observed that the strain–stress curves of all samples in Figure 8 can be divided into three regions, namely, elastic, plastic deformation, and strain hardening regions. The elastic region exhibits a linear change with recoverable deformation, followed by the plastic deformation region, in which neck forming would occur. The strain hardening region undergoes a strain hardening phenomenon with the continuous increase in the stress. It is clear that the Young’s modulus of the PBAT/MgO@CTAB nanocomposite increases with the increasing content of MgO@CTAB because MgO@CTAB particles serve as rigid fillers to transform the stress [30]. The tensile stress of PBAT nanocomposite achieves a maximum value of 29.90 MPa with the addition of 3 wt% MgO@CTAB, followed by a decreasing tendency due to the aggregation of MgO@CTAB, as revealed by SEM images. In addition, the elongation at break of PBAT nanocomposites increases slightly with the addition of a low content of MgO@CTAB. With the further increasing of nanofiller content, the elongation at break of PBAT decreases significantly. This is because the aggregation of MgO@CTAB causes stress concentration, which decreases the ductility.

**Figure 9:**
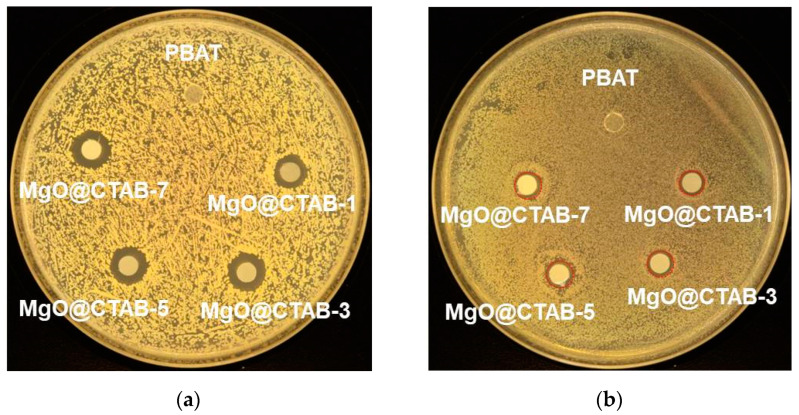
Photographs of antimicrobial test results of PBAT and its nanocomposite against (**a**) *S*. *aureus*, (**b**) *E*. *coli*.

The antimicrobial activity of the PBAT nanocomposite films against *S. aureus* and *E. coli* is shown in Figure 9, and the bacterial inhibition zone data are listed in Table 4. It is clear that no inhibition zone is observed for MgO@CTAB-0 film, indicating that pure PBAT film has no antibacterial activity against *S*. *aureus* and *E*. *coli*. The addition of MgO@CTAB endows the PBAT nanocomposite with antibacterial activity [10,14]. Moreover, the bacterial inhibition zone increases gradually with the increasing content of MgO@CTAB. These results can be ascribed to the superior antibacterial properties of MgO. However, PBAT/MgO@CTAB exhibits better antibacterial efficiency against *S*. *aureus* than against *E*. *coli*, which is similar to other studies in the literature [31,32,33,34]. This phenomenon is ascribed to the fact that the peptidoglycan membrane of the Gram-positive bacteria *S*. *aureus* is much thicker than that of the Gram-negative bacteria *E*. *coli*, and as a result, the released Mg ions do not easily penetrate the peptidoglycan membrane and disrupt the essential enzyme systems in the bacteria.

## 4. Conclusions

In this work, MgO@CTAB nanoparticles were synthesized and then mixed with a PBAT matrix using the melt compounding method. SEM observation and dynamical rheological measurement revealed that aggregation of MgO@CTAB occurred when the content of MgO@CTAB exceeded 5 wt%. PBAT nanocomposites containing 3 wt% MgO@CTAB exhibited the highest tensile strength of 27.90 MPa and a maximum elongation at break of 1773.95%. The presence of MgO@CTAB resulted in the poor thermal stability of PBAT due to its catalytic effect. In addition, MgO@CTAB enhanced the crystallinity of PBAT. The incorporation of MgO@CTAB endowed PBAT with antibacterial activity against both the Gram-positive bacteria *S. aureus* and the Gram-negative bacteria *E. coli.* The bacterial inhibition zone showed that PBAT/MgO@CTAB nanocomposites had better antibacterial performance against *S. aureus* than *E. coli.*

## Figures and Tables

**Table 1:** DSC thermograms of PBAT and its nanocomposites.

Samples	*T*_c_ (°C)	Δ*H*_c_ (J/g)	*T*_p_ (℃)	Δ*H*_m_ (J/g)	*χ* (%)
MgO@CTAB-0	97.0	8.1	125.3	9.2	8.1
MgO@CTAB-1	87.3	10.3	125.1	10.5	9.3
MgO@CTAB-3	89.0	11.1	124.5	11.0	9.9
MgO@CTAB-5	89.2	10.8	123.6	10.5	9.7
MgO@CTAB-7	90.8	9.9	122.5	10.0	9.4

**Table 2:** Thermal stability of PBAT and its nanocomposites.

Sample	*T*_10_ (°C)	*T*_p_ (°C)	Char Yield at 700 °C (wt%)
MgO@CTAB-0	375.2	400.7	6.67
MgO@CTAB-1	340.8	402.3	7.48
MgO@CTAB-3	328.3	399.9	10.32
MgO@CTAB-5	319.1	382.9	12.71
MgO@CTAB-7	313.7	381.2	14.02

**Table 3:** Tensile properties of PBAT nanocomposites.

Sample	Tensile Stress (MPa)	Young’s Modulus (MPa)	Elongation at Break (%)
MgO@CTAB-0	26.66 ± 2.27	43.37 ± 3.85	1590.73 ± 169.68
MgO@CTAB-1	27.06 ± 2.45	46.06 ± 2.49	1620.17 ± 107.53
MgO@CTAB-3	29.90 ± 2.01	48.81 ± 3.15	1773.95 ± 111.32
MgO@CTAB-5	25.03 ± 3.45	50.94 ± 2.15	1473.63 ± 116.27
MgO@CTAB-7	24.14 ± 1.33	54.93 ± 5.24	1398.33 ± 85.53

**Table 4:** Antimicrobial activity of PBAT nanocomposite films against *S. aureus* and *E. coli.*

Sample	Bacterial Inhibition Zone (mm)	
	*S. aureus*	*E. coli*
MgO@CTAB-0	5	5
MgO@CTAB-1	9.6 ± 0.6	5.9 ± 0.3
MgO@CTAB-3	10.3 ± 0.3	6.8 ± 0.5
MgO@CTAB-5	10.5 ± 0.3	7.1 ± 0.2
MgO@CTAB-7	10.7 ± 0.4	7.3 ± 0.2

## Data Availability

The raw/processed data required to reproduce these findings cannot be shared at this time as the data also forms part of an ongoing study.
